# Estimating the potential population impact of stepwise screening strategies for identifying and treating individuals at high risk of Type 2 diabetes: a modelling study

**DOI:** 10.1111/j.1464-5491.2012.03609.x

**Published:** 2012-07

**Authors:** P Chamnan, R K Simmons, K T Khaw, N J Wareham, S J Griffin

**Affiliations:** 1 MRC Epidemiology Unit, Institute of Metabolic ScienceCambridge, UK; 2 Clinical Gerontology Unit, University of Cambridge School of Clinical Medicine, Addenbrooke's HospitalCambridge, UK

**Keywords:** modelling study, population impact, prestratification, risk scores, screening, Type 2 diabetes

## Abstract

**Background:**

Diabetes risk assessment has been proposed as part of the National Health Service Health Checks programme, and HbA_1c_ has recently been recommended as a diagnostic test for diabetes at a threshold of 48 mmol/mol (6.5%). We estimated the potential population impact of different stepwise screening strategies to identify individuals at high risk who might be offered preventive interventions.

**Methods:**

Using data from 5910 participants in the European Prospective Investigation of Cancer (EPIC)-Norfolk cohort with HbA_1c_ measurements, we modelled different stepwise screening strategies for identifying and treating individuals at high risk of Type 2 diabetes using different HbA_1c_ cut-off points with and without a stage of prestratification. For each strategy, we estimated the number needed to have a diagnostic test, the number needed to treat to prevent one new case of Type 2 diabetes, and the number of new cases that could be prevented in the population over 3 years. Relative risk reductions for estimated effects of intensive lifestyle intervention were derived from the US Diabetes Prevention Program.

**Results:**

Compared with inviting all individuals in an average primary care trust for a diagnostic test, a stepwise screening approach using simple routine data such as age and anthropometric indices could prevent a slightly lower number (lower-upper estimates) of new cases of Type 2 diabetes over 3 years (224 [130–359] and 193 [109–315] cases respectively) but would only require half the population to be invited for a diagnostic blood test. A total of 162 (88–274) cases could be prevented by inviting individuals with a Cambridge risk score of ≥ 0.15, with only 40% of the total population requiring diagnostic blood tests. Using a participant completed questionnaire for risk assessment (FINDRISC) was less effective, mainly relating to the questionnaire response rate. Providing preventive interventions to those with a lower HbA_1c_ of 37–< 48 mmol/mol (5.5–< 6.5%) could prevent more cases but with a disproportionately higher workload, compared with using the recommended HbA_1c_ threshold of 42–< 48 mmol/mol (6.0–< 6.5%).

**Conclusions:**

Compared with mass screening, an approach using routine data for risk stratification followed by an HbA_1c_ test with a threshold of 42–< 48 mmol/mol (6.0–< 6.5%) for identifying individuals suitable for preventive interventions might prevent slightly fewer cases of Type 2 diabetes but with potential cost-savings.

## Introduction

Lifestyle and pharmacological interventions can halve the incidence of Type 2 diabetes (hereafter referred to as diabetes) in individuals with impaired glucose tolerance identified using an oral glucose tolerance test[1,2]. However, it is unlikely that population screening using an oral glucose tolerance test is a feasible method of identifying those at high risk in clinical practice [3,4] as it is time- and resource-consuming, and has poor reproducibility [5]. Population-based screening strategies for identifying individuals at high risk of diabetes need to be clinically, socially and ethically acceptable with a favourable risk-benefit balance [6].

Although previous studies suggest that interventions to prevent diabetes among individuals at high risk are likely to be cost-effective [4,7,8], few studies have investigated the benefits and costs of different screening strategies to identify those eligible for such preventive interventions [9,10]. No studies have accounted for activities and costs associated with identifying individuals at high risk who should be offered blood testing. No comparisons of strategies with and without prestratification using non-laboratory tests in the general population have been reported. There is consequently uncertainty about the potential efficiencies of using simple criteria or routine data before undertaking laboratory tests for blood glucose to identify individuals at high risk to whom preventive interventions might be offered [11].

Despite these uncertainties [12], the UK Department of Health recently introduced a national vascular risk assessment programme that includes stepwise screening for diabetes and impaired glucose tolerance. A diabetes risk score questionnaire (FINDRISC) has been recommended as a first step before inviting those at high risk for fasting plasma glucose (FPG) and oral glucose tolerance testing [13–15]. However, the FINDRISC has not been validated in a British population, and it requires individuals to complete and return self-reported questionnaires. An alternative approach might be to use routine data as a prestratification tool before a diagnostic blood test to identify those at high risk who should be offered preventive interventions. Using routinely available data does not necessitate the production, distribution and data entry of a questionnaire and, as we have shown for cardiovascular risk screening [16], may help reduce the number of diagnostic blood tests, thereby reducing the cost of the screening programme. This stepwise approach might also reduce the potential psychological harms which have been associated with screening tests, although for diabetes screening these appear to be limited [17,18].

The International Expert Committee [19] and the American Diabetes Association [20] suggest that individuals with an HbA_1c_ between 42 and 48 mmol/mol (6.0% and 6.5%) might represent a group in which the risk of development of diabetes is very high and which could therefore be targeted for individual preventive interventions. In addition, among non-diabetic individuals, HbA_1c_ has a stronger association with cardiovascular disease than fasting and 2-h post-load glucose [21], further justifying its incorporation into a vascular risk assessment programme [22]. There is a lack of evidence regarding the impact of using the newly proposed diagnostic test, with or without prestratification, for diabetes risk screening. We estimated the potential population impact of different stepwise screening strategies, including using routine data (simple information known to primary care practitioners, such as age and smoking status, or a previously validated simple risk score using data routinely available in primary care clinical records such as age, gender and prescribed medication), or self-reported diabetes risk questionnaire (for example, FINDRISC) as a first step followed by the use of HbA_1c_ to identify individuals at high risk who would subsequently be offered preventive interventions.

## Methods

### Study population

We used individual-level data from the European Prospective Investigation of Cancer-Norfolk (EPIC-Norfolk), a population-based prospective study that follows 25 639 men and women aged 40–74 years residing in the Norfolk region, UK. Details of the study have been described elsewhere [23]. Briefly, between 1993 and 1997, 77 630 individuals were invited from general practice to participate in the study. Of these, 25 639 (33%) consented and attended a baseline health assessment, where participants were questioned about their personal and family history of disease, medication and lifestyle factors such as smoking habits.

They were asked whether a physician had ever told them that they had any of the conditions in a list that included diabetes, heart attack and stroke. Anthropometric and blood-pressure measurements, and non-fasting blood samples were also taken. As funding for measurement of HbA_1c_ only became available in 1995, around half of all participants had information on this measure at baseline. The HbA_1c_ level was measured on fresh ethylenediaminetetraacetic acid (EDTA) blood samples using high-performance liquid chromatography (Diamat automated glycated haemoglobin analyser; Bio-Rad Laboratories Ltd, Hemel Hempstead, UK), which was standardized to the diabetes control and complications trial assay. Participants were invited to attend a second health assessment after 3 years (1998–2001), at which identical measurements were taken. The general practitioners of participants whose test results were abnormal [HbA_1c_≥ 53 mmol/mol (7.0%)] were notified so that they could assume responsibility for confirming diagnosis and arranging treatment. We limited our analyses to individuals with HbA_1c_ measurements at baseline and at the second health assessment (*n* = 6372). We also excluded those with missing data for other risk factors (e.g. age, sex, a family history of diabetes, smoking, the use of corticosteroids and anti-hypertensive drugs, BMI, waist circumference, systolic blood pressure) and one or more of the variables used to calculate diabetes risk scores (the Cambridge and Finnish risk scores), which would constitute a first step in stepwise screening (*n* = 294). We excluded 168 individuals with prevalent diabetes at baseline (clinically diagnosed diabetes), leaving 5910 individuals eligible for invitation for diabetes risk screening in our model. We further excluded 122 individuals with undiagnosed diabetes [baseline HbA_1c_≥ 48 mmol/mol (6.5%)] leaving 5,788 individuals for analysis.

### Definition of diabetes

We used data from baseline to the second health assessment to calculate the incidence of Type 2 diabetes over 3 years as previously reported [24]. Diabetes was defined clinically (self report, diabetes medication, diet modification, general practice or hospital diabetes registers or death certificates) and/or biochemically [HbA_1c_≥ 48 mmol/mol (6.5%)]. A capture–recapture analysis in this cohort indicated that using multiple sources of information for diabetes ascertainment detected 99% of total clinically incident cases, when compared with diagnostic information in a comprehensive review of medical records [25].

### Modelling screening strategies and subsequent preventive interventions

#### Screening stage

We modelled 12 different population-based stepwise screening strategies to identify individuals at high risk to whom preventive interventions would be offered. These can be divided into three main approaches: (1) a two-step screening including prestratification followed by an HbA_1c_ test with a threshold of 42–48 mmol/mol (6.0–6.4%); (2) a two-step screening including prestratification followed by an HbA_1c_ test with a threshold of 37–48 mmol/mol (5.5–6.4%); and (3) a single-step screening without an HbA_1c_ blood test. Details of each strategy are shown in Table 1.

**Table 1:** Strategies for identifying individuals at high risk who could be offered an intensive lifestyle intervention

Prestratification followed by HbA_1c_ 42–48 mmol/mol (6.0–6.4%)	
Strategy 1	Invite all individuals aged 40–74 years for a diagnostic blood test
Strategy 2	Invite all individuals aged 50–74 years for a diagnostic blood test
Strategy 3	Invite individuals aged 50–74 years who are overweight*
Strategy 4	Invite individuals aged ≥ 45 years with one or more risk factors for diabetes†
Strategy 5	High risk approach using a simple risk score incorporating routinely available data. Individuals would be ranked according to their CRS using data from electronic GP records. Individuals with a CRS value of ≥ 0.15 would be invited for a diagnostic blood test
Strategy 6	High risk approach using a participant completed questionnaire. Individuals with a score of ≥ 9 following completion of the FINDRISC questionnaire would be invited for a diagnostic blood test
Prestratification followed by HbA_1c_ 37–48 mmol/mol (5.5–6.4%)	
Strategy 7	Invite individuals aged 50–74 years who are overweight
Strategy 8	Invite individuals aged ≥ 45 years with one or more risk factors
Strategy 9	High risk approach using a simple risk score incorporating routinely available data (CRS ≥ 0.15).
Single-step screening without blood tests	
Strategy 10	Overweight individuals prescribed antihypertensive drugs
Strategy 11	Individuals aged ≥ 45 years with one or more risk factors
Strategy 12	Individuals with a CRS value of ≥ 0.50

CRS, Cambridge diabetes risk score; FINDRISC, Finnish diabetes risk score.

* Overweight = BMI ≥ 25 kg/m^2^ or waist circumference > 94 cm in men and > 80 cm in women

† Risk factors include obesity (BMI ≥ 30 kg/m^2^), family history of diabetes, or prescribed antihypertensive drugs; Australian recommendations [47].

For the two-step screening, individuals at high risk according to a prestratification step would be invited for a diagnostic blood test (HbA_1c_). It was assumed that 75% of people invited would attend the second step of screening by HbA_1c_ testing (Table 2). For single-step strategies based solely on non-laboratory risk factor information, we also assumed that 75% of people would attend for consideration of preventive interventions. In accordance with the recommendations by the International Expert Committee and the American Diabetes Association [19,20], individuals identified by the prestratification step who had an HbA_1c_ of 42–48 mmol/mol (6.0–6.4%) were considered as high risk and would be offered interventions to prevent diabetes (Strategies 1–6). We also modelled alternative strategies in which individuals with an HbA_1c_ of 37–48 mmol/mol (5.5–6.4%) (Strategies 7–9) and individuals defined as high risk according to single-step non-laboratory criteria (Strategies 10–12) would be offered preventive interventions.

**Table 2:** Assumptions used to model the potential population impact of different screening strategies and subsequent preventive interventions

Parameter	Estimate	95% CI or range in the sensitivity analysis	Reference
Prevalence of diabetes/HbA_1c_			EPIC-Norfolk
Screen-detected diabetes [HbA_1c_≥ 48 mmol/mol (≥ 6.5%)]	2.1%		
HbA_1c_ 42–<48 mmol/mol (6.0–6.4%)	6.7%		
HbA_1c_ 37–<42 mmol/mol (5.5–5.9%)	24.2%		
HbA_1c_ < 37 mmol/mol (< 5.5%)	69.1%		
Incidence of diabetes for each HbA_1c_ levels and different groups at risk			EPIC-Norfolk
Response to the FINDRISC questionnaire	60%	40%, 80%, 100%	
Response to screening invitation	75%	65–85%	Department of Health modelling [15]
Equal response rate to screening between each strategy and between all individuals invited			Department of Health modelling [15]
Prevention of diabetes			
Rate of uptake (pt)	85%	As low as 30%	Department of Health modelling [15], Ruge *et al*. [3]
Rate of adherence (pa)	90%	As low as 30%	Department of Health modelling [15], Ruge *et al*. [3]
RRR for prevention of diabetes (equivalent for groups with different risk)	0.58	0.48–0.66, as low as 0.2	US DPP [2]
The number of events (new cases of diabetes) that could be prevented in the population (NEPP)			Gemmell *et al*. [35]

FINDRISC, Finnish diabetes risk score. EPIC, European Prospective Investigation of Cancer. DPP, diabetes prevention program.

NEPP = *N* × pt × pa × (diabetes incidence) × RRR

where *N* = number of people eligible for intensive lifestyle interventions, RRR = relative risk reduction. Upper and lower estimates of the NEPP is calculated by applying point estimates of pt and pa to 95% confidence intervals associated with incidence and relative risk reduction.

#### Screening tools

We used the Cambridge risk score (CRS) and the FINDRISC as examples of a diabetes risk score using routine data from general practice and a self-reported diabetes risk questionnaire respectively. The CRS is a tool developed in a British population to identify people at risk of undiagnosed diabetes [26]. It was derived using data on age, sex, smoking, family history of diabetes, BMI and prescribed steroids and antihypertensive drugs, variables, which are increasingly routinely available in primary care. This simple risk score has been shown to predict prevalent undiagnosed diabetes in different ethnic groups [27], incident diabetes [28] and all-cause mortality [29].

The FINDRISC questionnaire is a self-completion diabetes risk score developed in a Finnish population [30], which has been validated in a number of countries [31,32], but not in the UK. The FINDRISC score is calculated using data on age, self-reported use of antihypertensive drugs, history of high blood glucose, physical activity of at least 4 h a week, daily consumption of vegetables, fruits and berries, and self-reported BMI and waist circumference. We assumed that the response rate to the self-administered FINDRISC questionnaire would be 60% (Table 2). We used a simple ordered index of overall physical activity derived from the baseline EPIC physical activity questions to denote whether participants engaged in at least 4 h of physical activity per week [33]. This index has previously been validated against heart rate monitoring [33] and is associated with incident cardiovascular disease and all-cause mortality in the EPIC-Norfolk cohort [34]. We used a simple seven-item diet question to examine whether EPIC participants consumed vegetables, fruit or berries daily. As data on past history of high blood glucose were not available in EPIC-Norfolk, we did not include this variable in the calculation of FINDRISC. We carried out a sensitivity analysis for different response rates to the FINDRISC questionnaire (40%, 80% and 100%) and for the FINDRISC cut-off points of 12 and 7, assuming that nobody had a history of hyperglycaemia and that all individuals had a history of hyperglycaemia, respectively.

#### Prevention stage

We assumed that high-risk individuals would be offered an intensive lifestyle intervention, similar to that described in the Finnish and USA diabetes prevention programmes [1,2]. We assumed the same relative risk reductions for people with different levels of diabetes risk. Table 2 shows the assumptions we made concerning rates of uptake, adherence and relative risk reductions for the lifestyle intervention.

We estimated the cumulative incidence of diabetes, the sensitivity and specificity and the prediction of incident diabetes, measured by the area under the receiver operating characteristic (ROC) curve, for each screening strategy (prestratification followed by HbA_1c_ testing). We further estimated the number needed to screen to prevent one new case of diabetes, the number needing to receive the intervention to prevent one new case of diabetes and the number of events (new cases of diabetes) that could be prevented in the population (NEPP) over 3 years [35]. For demonstration, we also calculated the NEPP for an average primary care trust with a catchment area including 136 900 people aged 40–74 years using mid-2007 population estimates [36]. We made a number of assumptions to model the population impact of different stepwise screening strategies and interventions (Table 2). We also modelled the population impact for scenarios in which (1) 65% and 85% of individuals invited would attend a further assessment, (2) more plausible rates of uptake and adherence to the intervention were assumed (30% uptake and adherence) or (3) the preventive intervention would be less effective than reported in the trials (relative risk reduction of 0.20 rather than 0.58). We also conducted a sensitivity analysis to examine whether the population impact of the strategies incorporating routine data (CRS) might change assuming that data on family history of diabetes, smoking, and/or BMI were unavailable.

## Results

Table 3 shows the characteristics of 5910 participants in the EPIC-Norfolk cohort by different HbA_1c_ categories and diabetes status. The mean age of participants was 57 (SD = 9) years, and 44% were male.

**Table 3:** Comparison of baseline characteristics across categories of baseline HbA_1c_ in 5910 participants in the European Prospective Investigation of Cancer (EPIC)-Norfolk cohort

		HbA_1c_ level					
			
	Total	< 31 mmol/mol (< 5.0%)	31–37 mmol/mol (5.0–5.4%)	37–42 mmol/mol (5.5–5.9%)	42–48 mmol/mol (6.0–6.4%)	≥ 48 mmol/mol (6.5%) (screen-detected diabetes)	P*
Number (%)	5910 (100)	1864 (31.5)	2138 (36.2)	1401 (23.7)	385 (6.5)	122 (2.1)	
Age, years	57.5 (9.4)	54.1 (9.1)	57.4 (9.1)	60.3 (8.7)	62.1 (8.2)	63.6 (7.6)	< 0.001
Men, *n* (%)	2590 (43.8)	753 (40.4)	946 (44.3)	638 (45.5)	183 (47.5)	70 (57.4)	< 0.001
Social class IIIb–V, *n* (%)	2087 (35.3)	587 (31.5)	767 (35.9)	527 (37.6)	157 (40.8)	49 (40.2)	< 0.001
Smoking, *n* (%)							< 0.001
Never	2945 (49.8)	994 (53.3)	1063 (49.7)	672 (48.0)	170 (44.2)	46 (37.7)	
Former	2426 (41.1)	728 (39.1)	912 (42.7)	569 (40.6)	159 (41.3)	58 (47.5)	
Current	539 (9.1)	142 (7.6)	163 (7.6)	160 (11.4)	56 (14.6)	18 (14.8)	
Family history of diabetes, *n* (%)	724 (12.3)	216 (11.6)	253 (11.8)	183 (13.1)	52 (13.5)	20 (16.4)	0.345
Use of corticosteroids, *n* (%)	166 (2.8)	44 (2.4)	61 (2.9)	38 (2.7)	14 (3.6)	9 (7.4)	0.019
Use of antihypertensive drugs, *n* (%)	869 (14.7)	194 (10.4)	300 (14.0)	250 (17.8)	85 (22.1)	40 (32.8)	< 0.001
BMI, kg/m^2^	26.0 (3.7)	25.4 (3.5)	25.9 (3.6)	26.3 (3.8)	26.8 (4.1)	28.2 (4.4)	< 0.001
BMI category, *n* (%)							< 0.001
< 25 kg/m^2^	2554 (43.2)	931 (50.0)	911 (42.6)	551 (39.3)	132 (34.3)	29 (23.8)	
25–29.9 kg/m^2^	2604 (44.1)	752 (40.3)	962 (45.0)	659 (47.0)	181 (47.0)	50 (41.0)	
>= 30 kg/m^2^	752 (12.7)	181 (9.7)	265 (12.4)	191 (13.6)	72 (18.7)	43 (35.3)	
Waist circumference, cm	87.2 (12.3)	84.9 (12.2)	87.2 (11.9)	88.6 (11.9)	90.7 (13.0)	96.3 (12.7)	< 0.001
Systolic blood pressure, mmHg	133.7 (17.6)	130.7 (17.1)	133.5 (17.2)	135.8 (18.2)	139.2 (17.5)	141.7 (16.7)	< 0.001
Diastolic blood pressure, mmHg	82.2 (10.9)	81.0 (10.6)	82.2 (10.8)	82.9 (11.2)	84.4 (11.1)	86.1 (10.9)	< 0.001
Total cholesterol, mmol/l	6.1 (1.1)	5.9 (1.1)	6.1 (1.1)	6.3 (1.1)	6.5 (1.3)	6.4 (1.1)	< 0.001
HDL cholesterol, mmol/l	1.5 (0.4)	1.5 (0.4)	1.5 (0.4)	1.4 (0.4)	1.4 (0.4)	1.3 (0.4)	< 0.001
Triglyceride, mmol/l, median (interquartile range)	1.5 (1.0–2.1)	1.3 (0.9–1.8)	1.5 (1.0–2.1)	1.6 (1.1–2.3)	1.8 (1.2–2.6)	2.0 (1.4–3.1)	< 0.001
Cambridge diabetes risk score, median (interquartile range)	0.10 (0.04–0.26)	0.06 (0.02–0.17)	0.10 (0.04–0.25)	0.14 (0.06–0.32)	0.19 (0.07–0.42)	0.39 (0.16–0.65)	< 0.001
FINDRISC, median (interquartile range)	8 (5–10)	6 (4–9)	7 (5–10)	8 (6–11)	9 (6–12)	11 (8–13)	< 0.001

FINDRISC, Finnish diabetes risk score. Data are presented in mean (standard deviation), unless specified otherwise.

* Differences between groups using *x*^2^ tests for categorical variables, and analysis of variance (anova) or Kruskal–Wallis tests for normally or non-normally distributed continuous variables.

### Incidence of Type 2 diabetes

Among the 5788 participants free of diabetes at baseline, 77 developed diabetes over 3 years. The cumulative incidence was 1.3% (95% CI 1.0–1.6) over 3 years, an annual incidence of 0.4%. Seven per cent of the study population had an HbA_1c_ of 42–48 mmol/mol (6.0–6.4%) at baseline; 38% of those who developed diabetes were in this group.

### Performance of different strategies in identifying individuals at high risk of Type 2 diabetes

The number of individuals that would need to be invited for a diagnostic HbA_1c_ test, the number of individuals with incident diabetes, the sensitivity/specificity and the predictive ability of each screening strategy are shown in Table 4. If individuals aged ≥ 50 years were invited to diabetes screening (Strategy 2), three-quarters of the total population aged 40–74 years would require blood tests and most cases of incident diabetes would be identified (88%). About half the population would need inviting for further assessment if age, BMI and waist circumference cut-off points were used for risk prestratification (Strategy 3), and this strategy would identify 74% of incident diabetes. The strategy using the presence of risk factors in individuals aged ≥ 45 years (Strategy 4), which required only one-third of the total population to undergo blood testing, would identify just over half of incident cases of diabetes. Strategies using the CRS cut-off point of ≥ 0.15 and a participant-completed FINDRISC questionnaire (Strategies 5 and 6) would require around 40% of the total population to be invited for blood tests. These two strategies identified around two-thirds of incident diabetes.

**Table 4:** Relative performance of different population screening strategies: number of individuals invited to screening for risk of diabetes, the incidence of diabetes over 3 years, sensitivity, specificity and the area under the receiver operating characteristic curve for prediction of incident diabetes

	Number of individuals invited to screening (%)	Incident cases of diabetes in risk group* (%)	Incidence of diabetes over 3 years, % (95% CI) in those invited	Sensitivity (95% CI) for prediction of incident diabetes over 3 years	Specificity (95% CI) for prediction of incident diabetes over 3 years	Area under the receiver operating characteristic curve (95% CI) for prediction of incident diabetes over 3 years
Prestratification followed by HbA_1c_ 42–48 mmol/mol (6.0–6.4%)						
Strategy 1: all individuals	5910 (100)	77 (100)	1.33 (1.04–1.63)	0.38 (0.27–0.49)	0.94 (0.93–0.94)	0.66 (0.60–0.71)
Strategy 2: age ≥ 50 years	4443 (75)	68 (88)	1.57 (1.20–1.94)	0.36 (0.26–0.48)	0.94 (0.94–0.95)	0.65 (0.60–0.71)
Strategy 3: age ≥ 50 years AND overweight†	2977 (50)	57 (74)	1.98 (1.47–2.49)	0.32 (0.22–0.44)	0.96 (0.95–0.97)	0.64 (0.59–0.70)
Strategy 4: age ≥45 years AND one or more of risk factor for diabetes^‡^	1868 (32)	42 (55)	2.34 (1.64–3.03)	0.23 (0.15–0.35)	0.97 (0.97–0.98)	0.60 (0.56–0.65)
Strategy 5: CRS ≥ 0.15	2361 (40)	49 (64)	2.16 (1.56–2.76)	0.27 (0.18–0.39)	0.96 (0.96–0.97)	0.62 (0.57–0.67)
Strategy 6: FINDRISC ≥ 9	2453 (42)	49 (64)	2.07 (1.50–2.65)	0.29 (0.19–0.40)	0.97 (0.96–0.97)	0.63 (0.57–0.68)
Prestratification followed by HbA_1c_ 37–48 mmol/mol (5.5–6.4%)						
Strategy 7: age ≥50 years AND overweight	2977 (50)	57 (74)	1.98 (1.47–2.49)	0.57 (0.45–0.68)	0.82 (0.81–0.83)	0.69 (0.64–0.75)
Strategy 8: age ≥45 years AND one or more of risk factor for diabetes	1868 (32)	42 (55)	2.34 (1.64–3.03)	0.44 (0.33–0.56)	0.89 (0.88–0.90)	0.66 (0.61–0.72)
Strategy 9: CRS ≥ 0.15	2361 (40)	49 (64)	2.16 (1.56–2.76)	0.51 (0.39–0.62)	0.85 (0.84–0.86)	0.68 (0.62–0.73)
Single-step screening without blood tests						
Strategy 10: overweight AND antihypertensive drugs	N/A	16 (21)	2.56 (1.32–3.80)	0.21 (0.13–0.32)	0.89 (0.89–0.90)	0.55 (0.50–0.60)
Strategy 11: age ≥45 years AND one or more of risk factor for diabetes	N/A	42 (55)	2.34 (1.64–3.03)	0.55 (0.43–0.66)	0.69 (0.68–0.70)	0.62 (0.56–0.68)
Strategy 12: CRS ≥ 0.50	N/A	18 (23)	3.27 (1.78–4.76)	0.23 (0.15–0.35)	0.91 (0.90–0.91)	0.57 (0.52–0.62)

CRS, Cambridge diabetes risk score; FINDRISC, Finnish diabetes risk score; N/A, not applicable.

* Risk group refers to individuals free of diabetes (i.e. 5788 individuals for Strategy 1). †Overweight: BMI ≥ 25 kg/m^2^ or waist circumference ≥ 94 cm in men and ≥ 80 cm in women. ^‡^Risk factors include BMI of ≥ 30 kg/m^2^, family history of diabetes and the use of antihypertensive drugs

Inviting individuals aged ≥ 50 years (Strategy 2) did not compromise the sensitivity, specificity and predictive ability for incident diabetes, compared with inviting all individuals (Strategy 1) (Table 4). Strategies inviting the overweight aged ≥ 50 years and those at high risk using diabetes risk scores (Strategies 3, 5 and 6) showed slightly lower sensitivity and discriminatory ability, compared with inviting all individuals (Strategy 1).

As 63% of incident cases of diabetes had a baseline HbA_1c_ of < 42 mmol/mol (6.0%), strategies using an HbA_1c_ cut-off point of 37–48 mmol/mol (5.5–6.4%) (Strategies 7–9) had higher sensitivity and better predictive ability, as measured by area under ROC curve, than strategies using an HbA_1c_ of 42–48 mmol/mol (6.0–6.4%) (Strategies 3–5). Strategies without blood tests (Strategies 10–12) appeared to have lower discrimination, compared with the two-step strategies including blood tests for HbA_1c_.

### The impact of screening strategies on the prevention of Type 2 diabetes in the population

Table 5 shows the potential population impact of the different stepwise screening strategies and subsequent treatment. Among strategies using an HbA_1c_ cut-off of 42–48 mmol/mol (6.0–6.4%), Strategy 1 would prevent the highest number of new cases of diabetes with a NEPP of 9.7, inviting those aged ≥ 50 years (Strategy 2) had a slightly lower NEPP value of 9.3. Inviting overweight individuals aged ≥ 50 years (Strategy 3) had a NEPP of 8.3, while the strategy using CRS (Strategy 5) had a greater impact than the strategy using a participant-completed FINDRISC questionnaire (Strategy 6) (NEPPs of 7.0 and 4.4, respectively), despite having a similar number needed to screen with HbA_1c_ and number needed to intervene to prevent one new case of diabetes. Strategies 3, 4 and 5 would need the fewest number of individuals to undergo testing for HbA_1c_ in order to prevent one new case of diabetes (just half that of Strategy 1), and these strategies would require fewer people to undergo treatment to prevent one new case of diabetes than Strategy 1 (16–18 vs. 23 individuals). When applying the NEPP to the population of an average primary care trust, we found that 224 new cases of diabetes could be prevented over 3 years if all individuals aged 40–74 years were invited for diabetes screening using HbA_1c_ testing, while 193 and 162 new cases could be prevented if only overweight individuals aged ≥ 50 years and those with a CRS of ≥ 0.15 were invited for diagnostic blood tests, respectively (50% and 40% of the total population, respectively). A strategy of inviting individuals aged ≥ 45 years with one or more risk factors for diabetes for HbA_1c_ testing (Strategy 4 – one-third of the total population invited) could prevent 139 new cases.

**Table 5:** Relative performance of different population screening strategies: population impact of screening for risk of diabetes in European Prospective Investigation of Cancer (EPIC)-Norfolk and subsequent treatment on prevention of diabetes (*n* = 5910)

	Number of people eligible for lifestyle interventions (% of total population)	Number needed to screen with HbA_1c_ to prevent one new case of diabetes	Number needed to intervene to prevent one new case of diabetes	NEPP for a population of 5910 people (lower and upper estimates)	NEPP for an average primary care trust with a catchment area of 136,900 people aged 40–74 years (lower and upper estimates)
Prestratification followed by HbA_1c_ 42–48 mmol/mol (6.0–6.4%)					
Strategy 1: all individuals	289 (5%)	459	23	9.7 (6.8–13.6)	224 (157–315)
Strategy 2: age ≥ 50 years	264 (4%)	358	22	9.3 (6.5–13.2)	216 (151–306)
Strategy 3: age ≥ 50 years AND overweight*	188 (3%)	268	17	8.3 (5.7–12.0)	193 (132–277)
Strategy 4: age ≥45 years AND one or more of risk factor for diabetes†	123 (2%)	234	16	6.0 (3.8–9.1)	139 (89–212)
Strategy 5: CRS ≥ 0.15	167 (3%)	253	18	7.0 (4.6–10.4)	162 (107–241)
Strategy 6: FINDRISC ≥ 9	99 (2%)	251	17	4.4 (2.9–6.5)	102 (68–150)
Prestratification followed by HbA_1c_ 37–48 mmol/mol (5.5–6.4%)					
Strategy 7: age ≥50 years AND overweight	818 (14%)	152	43	14.6 (9.7–21.8)	339 (226–505)
Strategy 8: age ≥45 years AND one or more of risk factor for diabetes	506 (9%)	124	34	11.3 (7.1–17.7)	262 (165–411)
Strategy 9: CRS ≥ 0.15	671 (11%)	136	40	13.0 (8.4–19.8)	301 (195–459)
Single-step screening without blood tests					
Strategy 10: overweight AND antihypertensive drugs	660 (11%)	N/A	67	5.3 (2.7–7.9)	123 (64–183)
Strategy 11: age ≥45 years AND one or more of risk factor for diabetes	1,868 (32%)	N/A	74	14.0 (9.8–18.2)	324 (227–421)
Strategy 12: CRS ≥ 0.50	599 (10%)	N/A	53	6.0 (3.3–8.7)	139 (76–202)

CRS, Cambridge diabetes risk score; FINDRISC, Finnish diabetes risk score; NEPP, the number of events (new cases of diabetes) that could be prevented in the population; N/A, not applicable

* Overweight: BMI ≥ 25 kg/m^2^ or waist circumference ≥ 94 cm in men and ≥ 80 cm in women.

† Risk factors include body mass index of ≥ 30 kg/m^2^, family history of diabetes and the use of antihypertensive drugs.

Stepwise strategies using an HbA_1c_ cut-off point of 37–48 mmol/mol (5.5–6.4%) could prevent twice as many cases of diabetes, compared with strategies using an HbA_1c_ cut-off point of 42–48 mmol/mol (6.0–6.4%), but necessitated offering interventions to three to five times as many people (more than twice as many people needed to undergo treatment to prevent one new case of diabetes). Strategies without blood tests (Strategies 10 and 12) could prevent a considerably lower number of new cases of diabetes than strategies using HbA_1c_ testing (Strategies 8 and 9) when an intervention was provided to 10% of the population in each case. Although inviting individuals with one or more risk factors for lifestyle interventions (Strategy 11) was among the strategies that could prevent the highest number of new cases of diabetes, the strategy had the highest number needed to intervene/treat.

In a sensitivity analysis, when the response rate to the participant completed questionnaire (FINDRISC) was 40%, the number of new cases of diabetes that could be prevented was 68 and, when a perfect response rate of 100% was achieved, this strategy could prevent 170 new cases of diabetes, which is comparable to the strategy using the CRS (Strategy 5). Using a FINDRISC cut-off point of ≥ 7 (assuming all individuals had a history of hyperglycaemia) identified 78% of those who developed diabetes over 3 years, and this approach could prevent 116 new cases of diabetes. Using a FINDRISC cut-off point of ≥ 12 (equivalent to the situation in which nobody had a history of hyperglycaemia) identified only 40% of those who developed diabetes, and this strategy had the lowest NEPP; only 60 new cases could be prevented. The number of new cases of diabetes that could be prevented when attendance rates were changed from 65% to 85% increased from 194 to 253 cases for the strategy inviting all individuals, from 167 to 218 cases for the strategy using simple information on age and anthropometry (Strategy 3) and from 140 to 183 cases for the strategy using a simple risk score (CRS).

The population impact of screening strategies and subsequent lifestyle interventions was very sensitive to changes in the uptake, adherence and effectiveness of interventions. For example, a decrease in rates of uptake and adherence, and relative risk reduction of an intensive lifestyle intervention led to proportionate reductions in the population impact of all screening strategies (data not shown). Lastly, excluding family history of diabetes, smoking and/or BMI from the CRS did not significantly reduce the population impact of the strategies using routine data as a prestratification tool.

## Discussion

### Summary of findings

We estimated the potential population impact of different stepwise screening strategies for identifying and treating individuals at high risk of diabetes based on different combinations of prestratification and HbA_1c_ cut-off points. Compared with mass screening, stepwise screening strategies incorporating simple criteria or routine data as a first step before inviting those at high risk for a diagnostic blood test and subsequent preventive interventions could prevent slightly fewer new diabetes cases but would greatly reduce the number of diagnostic blood tests and number of individuals receiving lifestyle interventions. A stepwise strategy incorporating a participant completed questionnaire (for example FINDRISC) would prevent fewer new cases of diabetes, although this was mainly dependent on the response rate to the questionnaire. Lifestyle interventions for individuals with an HbA_1c_ of 37–48 mmol/mol (5.5–6.4%) could prevent more new cases of diabetes, but with disproportionately higher workload (costs), compared with the recommended HbA_1c_ cut-off of 42–48 mmol/mol (6.0–6.4%). Single-step screening using simple criteria or routine data without blood tests was less effective at preventing diabetes than two-step screening incorporating blood tests for HbA_1c_ when similar proportions of the population were eligible for preventive interventions.

### Comparison with previous studies

A few previous studies have examined the cost-effectiveness of screening for diabetes and glucose intolerance using fasting plasma glucose and oral glucose tolerance testing [9,10,37]. Using different sophisticated modelling techniques, these studies suggest that screening for diabetes and impaired glucose tolerance followed by prevention interventions was likely to be cost-effective, compared with the National Institute for Health and Clinical Excellence (NICE) ‘willingness to pay’ threshold of £20 000 per Quality-adjusted life year (QALY) gained [38]. However, the service costs of the diagnostic tests and preventive interventions used in the above studies varied a great deal and may have been underestimated, compared with other modelling studies [39]. Furthermore, the authors did not consider targeted screening in their analysis. That is, no comparisons of strategies with and without prestratification using non-laboratory tests were reported. Targeted screening using multivariate diabetes risk scores has previously been shown to be effective at identifying individuals with undiagnosed and incident diabetes in several populations [26,27,30]. This approach, particularly risk scores incorporating routine data or easily-measured risk factors, may help refine a subgroup in the population that requires further blood testing, which might in turn reduce costs of the screening programme.

Diabetes screening identifies individuals with both prevalent undiagnosed diabetes and those at high risk of developing diabetes (i.e. impaired fasting glucose and impaired glucose tolerance). The previous cost-effectiveness studies addressed different target groups, making it difficult to compare the outcomes across studies. Our analysis focused on strategies for identifying and treating individuals at high risk of developing diabetes, which is mainly concerned with prevention and not screening. However, policy decisions should also be informed by the potential benefits of early detection of prevalent but undiagnosed diabetes.

A modelling study commissioned by the Department of Health suggested that stepwise screening and subsequent interventions would prevent 4000 new cases of diabetes each year. The authors used the FINDRISC questionnaire as a prestratification tool before inviting those with the FINDRISC score of ≥ 12 for a series of diagnostic blood tests (i.e. fasting plasma glucose and then oral glucose tolerance testing) [13]. However, this approach requires new data collection. Furthermore, a response rate to the questionnaire of 100% was assumed. We have demonstrated that the performance of this approach relies heavily on the response rate to a participant completed questionnaire, which in turn supports the use of routine data as a prestratification tool. Furthermore, experience from the Diabetes, Heart Disease and Stroke (DHDS) Pilot study suggested that sending out invitation letters or questionnaires is costly in terms of staff time [40]. This contrasts with strategies that use data routinely available in the health-care system, or simple patient information such as age and body mass index, to identify individuals at high risk who could be offered diagnostic blood testing. Beyond the necessity of new data collection, the Department of Health study used different cross-sectional datasets to generate a hypothetical population for modelling, while we used data from a single existing population-based prospective British cohort and measured actual diabetes incidence over 3 years.

### Strengths and limitations

Conclusions from modelling studies depend on the accuracy of the model and its underlying assumptions and time-frame [41], as well as setting-specific limitations which can influence the generalizability of the analysis, such as characteristics of health-care systems and the target population [8]. In contrast to previous studies which defined individuals at high risk using fasting plasma glucose or an oral glucose tolerance test, our study describes the benefits of stepwise population-based screening to identify high-risk individuals using an HbA_1c_ diagnostic test and subsequent preventive interventions. Our modelling was based on actual incidence of diabetes over 3 years with associated 95% confidence intervals in individuals with different levels of HbA_1c_ in a representative British population. Key assumptions were based on evidence from randomized control trials and sources of uncertainty were tested in sensitivity analyses. However, using a single-point estimate for rates of uptake and adherence to preventive interventions may underestimate the true uncertainty of the population impact. Lower rates of uptake and adherence to the intensive lifestyle interventions in a real-life setting might result in a lower population impact than our estimates, although the relative difference in the population impact between strategies would not be altered.

Participants included in this analysis had a more favourable risk factor profile than those who were excluded (mainly those without HbA_1c_ results because of the inclusion of HbA_1c_ later in the study when funding became available). Therefore, the incidence of diabetes and hence population impact measured in this study may be underestimated. Given the 33% recruitment rate in this study, it is possible that participants might be more health conscious and more likely to engage in healthy behaviours, compared with non-participants. However, EPIC-Norfolk participants were similar to the English population for most characteristics, such as anthropometric indices, blood pressure and serum lipid levels [23]. It is also unlikely that individuals at different levels of risk of developing diabetes have the same attendance rate. People at high risk are less likely to attend for screening (e.g. obese males are less likely to attend for screening than non-obese females) [42]. However, while this assumption may affect the number of diabetes cases that could be prevented, it is unlikely to alter the main finding that prestratification using routine data is more efficient than inviting all middle-aged individuals for diagnostic blood tests.

A relatively short follow-up period and relatively small number of incident cases mean that the findings from this analysis should be interpreted with caution. While one might be interested in long-term risk (e.g. traditional estimation of 10-year cardiovascular disease risk or lifetime risk) and benefits from preventive interventions (e.g. QALYs gained), this requires sophisticated modelling with a number of assumptions. Furthermore, the 3-year follow-up is still a plausible and important time frame for identifying those at high risk of diabetes as individuals are likely to be interested in information on short-term risk of diabetes and potential short-term benefits, and perhaps are more likely to change behaviour as a result of receiving this information [43].

In our modelling study, we only included individuals with complete data for calculating the risk scores, which might have influenced our results. However, general practices have relatively high levels of data completeness for smoking and BMI [44], and if no BMI is recorded this information can be obtained by using imputation techniques or from self-reported height and weight with reasonable accuracy [45]. In addition, the exclusion of such information did not alter the potential population impact of the strategies using routine data for prestratification. This suggests that this approach can be used in health systems where data on these variables are less readily available.

Overall, the sensitivity of the screening strategies that we modelled was low. Those who are not offered a screening test after a negative screening result might be affected by other psychological symptoms such as insecurity and anxiety about the chance of developing diabetes [46]. However, the performance of strategies using routinely available data for risk stratification was comparable with inviting all individuals for a diagnostic HbA_1c_ test. The sensitivity and specificity of the strategies in our study were also comparable with strategies combining the presence of risk factors and fasting plasma glucose or an oral glucose tolerance test for identifying individuals with glucose intolerance in studies in Australia and in Leicester, UK [40,47]. In fact, there are limited harms associated with diabetes screening [17] and little evidence of false reassurance associated with negative screening test results for diabetes [18]. Furthermore, the relatively modest discriminatory ability (area under ROC curve) for stepwise strategies compared with the published studies of risk scores might result from the fact that most have relied on a clinical diagnosis and not included biochemical testing to identify biochemically but not clinically incident cases. This leads to overestimation of the area under the ROC curve as the variables used to predict risk are similar to those that might lead practitioners to test for diabetes. It might also be explained by the incorporation of HbA_1c_ in strategies for predicting incident diabetes defined using multiple methods of diagnosis (e.g. use of fasting blood glucose in clinical practice). However, this impact might be limited as all individuals had HbA_1c_ results at follow-up in this modelling study.

It remains unclear whether intensive lifestyle interventions in those with impaired glucose tolerance would be similarly effective in individuals at high risk identified through different strategies, or in larger groups of the population with lower risk than those participating in trials. Lastly, as 99% of the EPIC-Norfolk participants are white, the generalizability of our findings to other ethnic groups is limited.

### Balance between benefits, harms and costs

Optimal choices for population-wide diabetes risk screening should be based on the balance between benefits, harms and costs. Inviting all middle-age individuals for a diagnostic blood test would be costly and time-consuming, while attempts to assess an individual’s risk of developing diabetes using questionnaire-based risk scores (such as the FINDRISC approach) might be hampered by low response rates to the questionnaire. Furthermore, as non-responders to the questionnaire tend to be less healthy, the real benefit of this approach is likely to be lower than our estimates. It is noteworthy that, in terms of diabetes prevention in this population, using diabetes risk scores for prestratification was not superior to the use of simple routine data such as age and the presence of other risk factors. Stepwise strategies using routine data for prestratification might help reduce the number of individuals who require blood tests and hence the costs of the screening programme [48]. Furthermore, as diabetes and cardiovascular disease share many common risk factors including HbA_1c_, combined diabetes and cardiovascular risk assessment in primary care might represent a more cost-effective approach to risk stratification [22].

The sensitivity and specificity of screening strategies will influence their relative impact on identifying those at high risk and hence preventing diabetes. Strategies with high sensitivity will correctly identify most individuals at high risk, but they will lead to unnecessary diagnostic tests and inefficient use of scarce preventive resources. On the other hand, strategies with high specificity will correctly identify those who should not be offered diagnostic tests and preventive interventions, but will overlook some individuals at high risk who could benefit from these interventions. Furthermore, decision-making on the optimal choice of stepwise diabetes risk screening will be informed by relative costs of diagnostic testing (e.g. facility, training, equipment, laboratory tests and transportation) and intensive lifestyle interventions (such as the relatively expensive Finnish or US diabetes prevention programmes). The difference in costs between the strategies proposed would be substantial, particularly if confirmation of diabetes diagnosis requires repetition of HbA_1c_ tests. This further supports the stepwise screening approach. Our study also suggests that efforts to enhance the uptake and compliance of intensive lifestyle interventions are necessary, and that development of effective preventive interventions applicable to a broader ‘real-world’ setting is important.

## Conclusions

In conclusion, our study has demonstrated the potential for using routine data for Type 2 diabetes risk prestratification before inviting individuals at high risk for a diagnostic HbA_1c_ test and subsequent preventive interventions. Compared with universal screening, using routine data for identifying those who should be offered diagnostic blood testing, followed by preventive interventions in those with an HbA_1c_ of 42–48 mmol/mol (6.0–6.4%) could prevent a significant number of Type 2 diabetes cases with potential cost-savings. A stepwise strategy incorporating a participant completed diabetes risk questionnaire (UK government recommendation) would be less effective, and this was mainly reliant on the response rate to the questionnaire. Use of routine data might therefore represent a feasible alternative for identifying individuals or groups to whom prevention interventions could be targeted. Providing preventive interventions to those with a lower HbA_1c_ of 37–48 mmol/mol (5.5–6.4%) may increase workload and costs disproportionately to the increased benefits of prevention of new cases of diabetes. However, primary research into the cost-effectiveness of different approaches for identifying and treating people at high risk of Type 2 diabetes is needed.
